# Hypoxia induces connexin 43 dysregulation by modulating matrix metalloproteinases via MAPK signaling

**DOI:** 10.1007/s11010-013-1793-5

**Published:** 2013-09-04

**Authors:** Xianghong Wu, Wen Huang, Gang Luo, Laval Andy Alain

**Affiliations:** 1 Department of Cardiology, First Affiliated Hospital, Guangxi Medical University, Nanning, People’s Republic of China; 2 Department of Neurology, First Affiliated Hospital, Guangxi Medical University, Nanning, 530021 People’s Republic of China

**Keywords:** Connexin 43, Matrix metalloproteinases, Mitogen-activated protein kinases, Hypoxia

## Abstract

Connexin 43 (Cx43) is a major structural protein found in the gap junctions of the ventricular myocardium and a major determinant of its electrical properties. The effects of matrix metalloproteinases (MMPs), the mitogen-activated protein kinase (MAPK) signaling pathway, transcription factor NF-kB, and activator protein-1 (AP-1)/c-Jun on the regulation of Cx43 gene expression in H9c2 cardiomyocytes were assessed. The MAPK signaling pathway (MEK/ERK1/2 and PI3K) and transcription factors NF-kB and AP-1/c-Jun were inhibited, then Cx43 expression was assessed using Western blot analysis, and MMP-9 activity was assessed using gelatin zymography. Hypoxia decreased the Cx43 protein level by approximately 30–50 %. Doxycycline (10 μg/mL), an inhibitor of MMP, markedly attenuated the hypoxia-induced downregulation of Cx43 protein expression at 6 h. The hypoxia-induced decrease in Cx43 protein expression was significantly reversed by U0126 (10 μM), a MEK/ERK1/2 inhibitor, at 6 and 12 h; LY294002 (30 μM), a PI3K inhibitor, downregulated Cx43 expression. Hypoxia-induced MMP-9 activation was inhibited by treatment with LY294002, U0126, and, most especially, U0126. JSH-23 (30 μM), an NF-kB inhibitor, and SP600125 (10 μM), an AP-1/c-Jun inhibitor, attenuated the loss of Cx43. These results suggest that MAPK signaling and the activities NF-kB and MMPs play an important roles in the regulation of Cx43 expression.

## Introduction

Gap junctions, which are composed of channel-forming integral membrane proteins known as connexins, mediate cell–cell communication in almost all tissues [1]. Connexin 43 (Cx43) is a major structural protein found in the gap junctions of the ventricular myocardium and a major determinant of myocardial electrical properties [2]. Cx43 dysfunction in cardiomyocytes may contribute to the pathogenesis of ventricular arrhythmias. Transferring the Cx43 gene to pigs with anterior infarction reduces ventricular tachycardia in the border zone of the healed scar [3].

Matrix metalloproteinases (MMPs) reportedly play an important role in the degradation of the extracellular matrix (ECM) [4]. The degradation of the ECM by MMPs is involved in the pathogenesis of cardiovascular diseases, including atherosclerosis and myocardial infarction [5, 6]. MMP expression is regulated at the transcription level by the activation of various transcription factors, such as activator protein (AP)-1 and nuclear factor kappa-light-chain enhancer of activated B cells (NF-κB) [7–9]. MMP-9 is expressed within minutes of myocardial ischemia [10]. MMPs are also capable of mediating Cx43 cleavage. Myocardial ischemia-induced reduction in Cx43 expression is also attenuated in MMP-7^−/−^ mice [11]. Our previously reported study found that lipopolysaccharide (LPS)-induced MMP-2 activation is affected by NF-κB-dependent Ras-MEK1/2 signaling [12]. Serine/threonine protein kinase B (PKB/Akt)—a major downstream effector of activated phosphoinositide-3 kinase (PI3K) and the ERK1/2 pathway—influences the transcription of MMP-9 [13]. HIV-1 induces the dysregulation of tight junction proteins by modulating MMPs via the ERK and Akt signaling [14, 15]. But, are MMPs and mitogen-activated protein kinase (MAPK) signaling involved in the regulation of gap junctions in cardiomyocytes, as they are in tight junctions? This present study was performed to determine if MMPs and MAPK signaling are involved in the hypoxia-induced regulation of Cx43. This study was performed to help to better understand the mechanisms involved in the development of ventricular arrhythmia following myocardial ischemia, as well as provide data that could be used to pharmacologically prevent ventricular arrhythmia in patients with a history of myocardial infarction.

## Methods

### Cell culture

Rat H9C2 cardiomyocytes were obtained from the Chinese Academy of Sciences (Beijing, China) and cultured in Dulbecco’s modified Eagle’s medium (DMEM; Sigma, St. Louis, MO, USA) supplemented with 10 % fetal bovine serum (FBS; Hyclone) and penicillin plus streptomycin (100 U/mL and 100 μg/mL, respectively). All cultures were maintained at 37 °C in a 5 % CO_2_ atmosphere.

### Hypoxic conditions and chemical treatment

Cells were incubated under hypoxic conditions to mimic in vivo myocardial ischemia. Hypoxic conditions were achieved in an incubator containing a gaseous mixture of 94 % N_2_, 5 % CO_2_, and 1 % O_2_ (v/v).

Cells were incubated overnight in serum-free essential medium before treatment with the indicated agents. In brief, H9C2 cardiomyocytes were cultured in 6-well plates until 90 % confluence, then treated under hypoxic conditions in the presence or absence of the following inhibitors: MMP inhibitor doxycycline (10 μg/mL; Sigma); PI3K inhibitor LY294002 (30 μM; Sigma); and MEK/ERK1/2 inhibitor U0126 (10 μM; Sigma). In specific experiments, H9C2 cells were treated with the NF-κB inhibitor JSH 23 (30 μM; Santa Cruz) or AP-1/c-Jun inhibitor SP600125 (10 μM; Sigma) for 6 or 12 h. Control cells were cultured in complete medium under normoxic conditions. After treatment, cells were harvested and assessed using Western blot analysis, and the culture medium was assayed for MMP-9 activity.

### Cell viability assay

Relative cell viability was determined using 125 mg/mL MTT (3-[4,5-dimethylthiazolyl-2]-2,5-diphenyltetrazolium bromide in phosphate-buffered saline) [16], which was added after treatment. Cells were incubated in MTT for 3 h at 37 °C and solubilized in dimethyl formamide (50 %; v/v) and sodium dodecyl sulfate (SDS) (20 %; w/v), and then absorbance was measured at 570 nm.

### Western blot analysis

Proteins from the H9C2 cells that were cultured in the 6-well plates were extracted using RIPA lysis buffer (Santa Cruz Biotechnology) and centrifuged at 15,000 *g* for 15 min. The supernatants were collected, and protein concentrations were determined using the BCM Protein Assay Kit (Thermo Scientific Pierce). An aliquot of 30 μg proteins from each sample was separated on 10 % Tris–HCl SDS-polyacrylamide gels, transferred onto nitrocellulose membranes, incubated with 3 % skim milk in Tris-buffered saline solution for 1 h, incubated overnight with the respective antibodies at 4 °C, and finally incubated with the peroxidase-conjugated secondary antibody at room temperature for 120 min. To visualize the proteins, the immunoblots were analyzed using the ECL Western blotting detection kit (Thermo Scientific Pierce). Rabbit anti-rat Cx43 antibodies were purchased from Abcam. Anti-ERK1/2, phosphorylated ERK1/2 (p-ERK1/2), PI3K, and phosphorylated PI3K (p-PI3K) antibodies were purchased from cell signaling. HRP-GAPDH was purchased from Kangcheng Biotechnology. All secondary antibodies were purchased from Santa Cruz Biotechnology.

### MMP-9 activity assay

MMP-9 activity was assessed by gelatin zymography [12] using premade 10 % polyacrylamide gels containing 0.1 % gelatin and 10 μL serum-free media from the treated cultures; these procedures were performed according to the instructions provided by the manufacturer (Invitrogen). Briefly, cells in serum-free medium were pretreated with 30 μM LY294002 or 10 μM U0126 for 2 h, followed by coexposure to hypoxia and specific inhibitors for 6 or 12 h. After electrophoresis, the gel was removed and incubated with Renaturing Buffer for 30 min at room temperature with gentle agitation, then equilibrated overnight with Developpin Buffer. Bands were visualized by staining for 30–60 min with 0.1 % Coomassie R-250 in 40 % ethanol and 10 % acetic acid, followed by distaining for 2 h at room temperature with a solution containing 10 % ethanol and 7.5 % acetic acid.

### MMP-9 promoter activity assays

H9C2 cells in 24-well plates were transfected with 0.5 μg pGL3 MMP-9 using lipofectin (Invitrogen) as the transfection reagent. To normalize to transfection efficiency, cells were cotransfected with 0.05 μg pRL-TK construct (Promega), which encodes for *Renilla* luciferase. Firefly and *Renilla* luciferase activities were determined using the Dual-Luciferase Reporter Assay System (Promega), as previously described [13].

### Statistical analysis

The intensities of the bands corresponding to specific proteins were determined using image J software. Routine statistical analyses were completed using SPSS 15.0. Values are presented as the mean ± SEM. One-way ANOVA was used to evaluate between-group differences, followed by the Tukey test. In this study, *p* < 0.05 is considered significant.

## Results

### MMPs and MAPK signaling are involved in Cx43 regulation

**Fig. 1:**
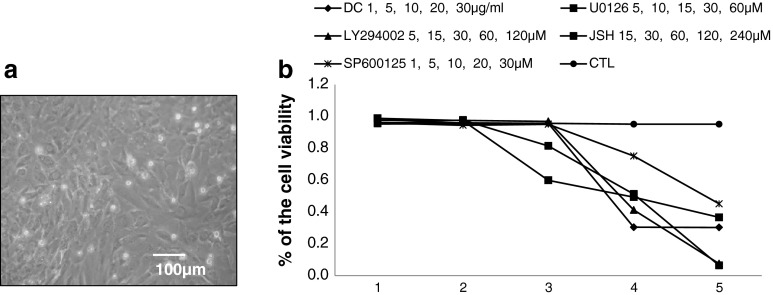
**a** Photomicrographs of the H9C2 cardiomyocytes. **b** Inhibitors on cell viability determined with MTT assays. H9C2 cells were treated with inhibitors for 12 h at different concentrations (doxycycline: 1, 5, 10, 20, and 30 μg/ml; LY294002: 5, 15, 30, 60, and 120 μM; U0126: 5, 10, 15, 30, and 60 μM; JSH-23: 15, 30, 60, 120, and 240 μM; SP600125: 1, 5, 10, 20, and 30 μM)

Rat H9C2 cardiomyocytes are shown in Fig. 1a. None of the chemical treatments affected cell viability at the concentrations that were used in the present study (Fig. 1b).

**Fig. 2:**
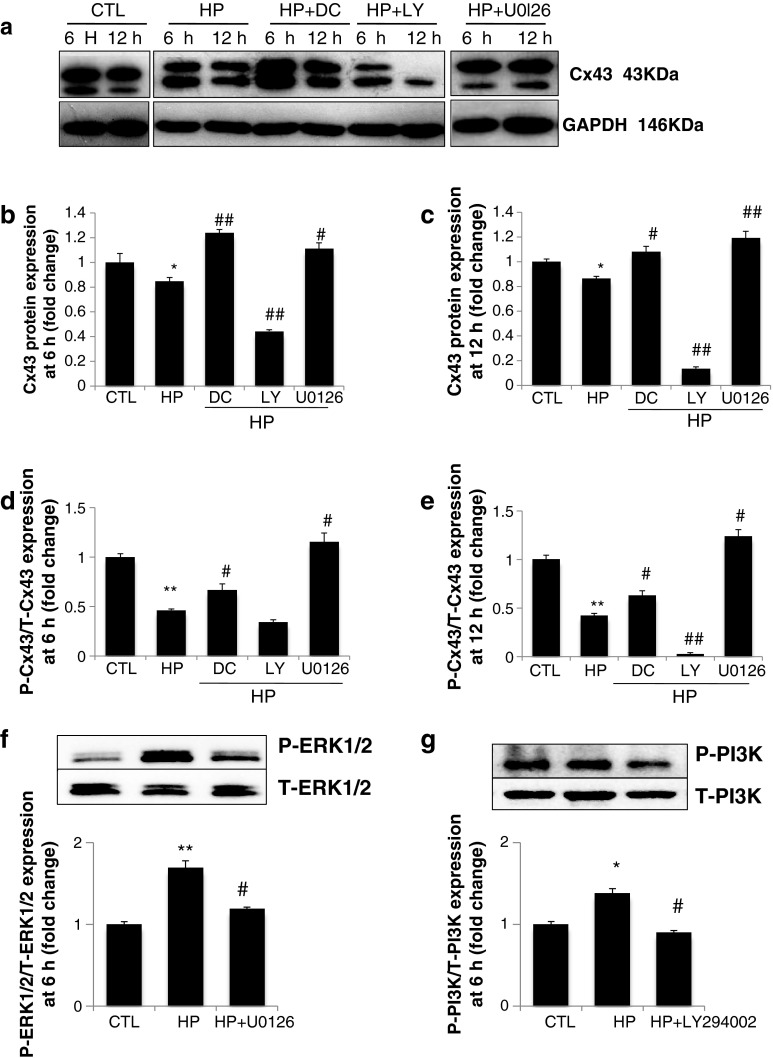
**a** Showing Western Blot band of Cx43 with treatment of hypoxia in the absence or presence of MMPs inhibitor doxycycline (10 μg/ml) or PI3K inhibitor LY294002 (30 μM) or MEK/ERK 1/2 inhibitor U0126 (10 μM) at 6 h and 12 h. **b–g** The quantified results are depicted in the form of bar graphs. Cx43 protein expression with treatment for 6 h (**b**) and 12 h (**c**). Hypoxia decreased the total Cx43 protein level by approximately 30–50 %. Doxycycline or U0126 markedly attenuated hypoxia-induced downregulation of Cx43 protein expression. LY294002 acts synergistically with hypoxia to downregulate Cx43 protein expression. The ratio of P-Cx43 to T-Cx43 is shown in (**d–e**). p-ERK1/2 and p-PI3K levels in response to U0126 and LY294002 were shown in **f–g**. Results represent the mean ± SEM of 3 separate experiments. (*Asterisk*) Significantly different as compared to the control. (*Hash*) Data in the groups co-treated with hypoxia and specific inhibitor are significantly different from those treated with hypoxia alone

MMPs are involved in the cleavage of Cx43. Administering MMP-2 to normal rats decreases Cx43 expression by 40 % [17]. To investigate the effects of MMPs on the expression of Cx43, H9C2 cells were treated with doxycycline, an inhibitor of MMPs. As illustrated in Fig. 2, hypoxia decreased the total Cx43 protein level by approximately 30–50 %. Doxycycline markedly attenuated the hypoxia-induced downregulation of total Cx43 protein expression at both 6 and 12 h, but most especially at 6 h. Our data indicate that MMPs are involved in the regulation of Cx43 protein expression, which is consistent with previous studies [17].

Our previously reported data indicate that the Akt and ERK1/2 pathways influence MMP-9 expression [14, 15]. To determine if MAPK signaling is involved in the regulation of Cx43 expression, the inhibitors LY294002 and U0126 were used to block PI3K and the MEK/ERK signaling pathway, respectively. The hypoxia-induced reduction in Cx43 protein expression was significantly reversed by U0126 at 6 and 12 h (Fig. 2a–c). Interestingly, the PI3K inhibitor LY294002 did not protect against hypoxia-induced alterations in Cx43. On the contrary, LY294002 acts synergistically with hypoxia to downregulate Cx43 protein expression.

The polyclonal Cx43 antibody may recognize several forms of phosphorylated Cx43 (p-Cx43). As shown in Fig. 2, lower bands correspond to low-molecular-weight proteins that are weakly phosphorylated, and upper bands correspond to high-molecular-weight proteins that are strongly phosphorylated [18]. Notably, MAPK signaling affected Cx43 phosphorylation. The ratio of p-Cx43 to total Cx43 proteins (T-Cx43) was significantly higher following exposure to U0126 and hypoxia in comparison with cells that were treated with hypoxia alone; however, a weaker phosphorylated form of Cx43 was induced with LY294002 under hypoxia (Fig. 2d–e). Meanwhile, p-ERK1/2 and p-PI3K levels were markedly upregulated in response to hypoxia and downregulated in response to U0126 and LY294002 (Fig. 2f–g).

### ERK1/2 signaling regulates Cx43 expression by modulating MMP-9 enzyme activity

**Fig. 3:**
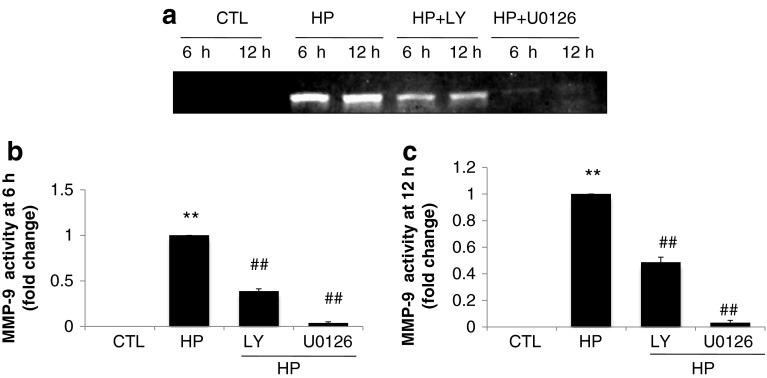
**a** Showing Zymography band of Cx43 with treatment of hypoxia in the absence or presence of LY294002 (30 μM) or U0126 (10 μM) at 6 h and 12 h. **b–c** The quantified results are depicted in the form of bar graphs. Cx43 protein expression with treatment for 6 h (**b**) and 12 h (**c**). Results represent the mean ± SEM of 3 separate experiments. Significantly different as compared to the control. (*Hash*) Data in the groups co-treated with hypoxia and specific inhibitor are significantly different from those treated with hypoxia alone

To determine if MMP-9 enzyme activity is involved in MAPK signaling and the regulation of Cx43, LY294002 (PI3K inhibitor) and U0126 (MEK/ERK inhibitor) were applied, respectively, and MMP-9 activity was measured using zymography. The zymography media were obtained from the same cultures used in the Western blot analysis. As shown in Fig. 3, hypoxia-induced MMP-9 activation was inhibited by treatment with LY294002 and U0126 at 6 and 12 h, especially U0126.

### NF-κB and AP-1/c-Jun are involved in the regulation of Cx43

**Fig. 4:**
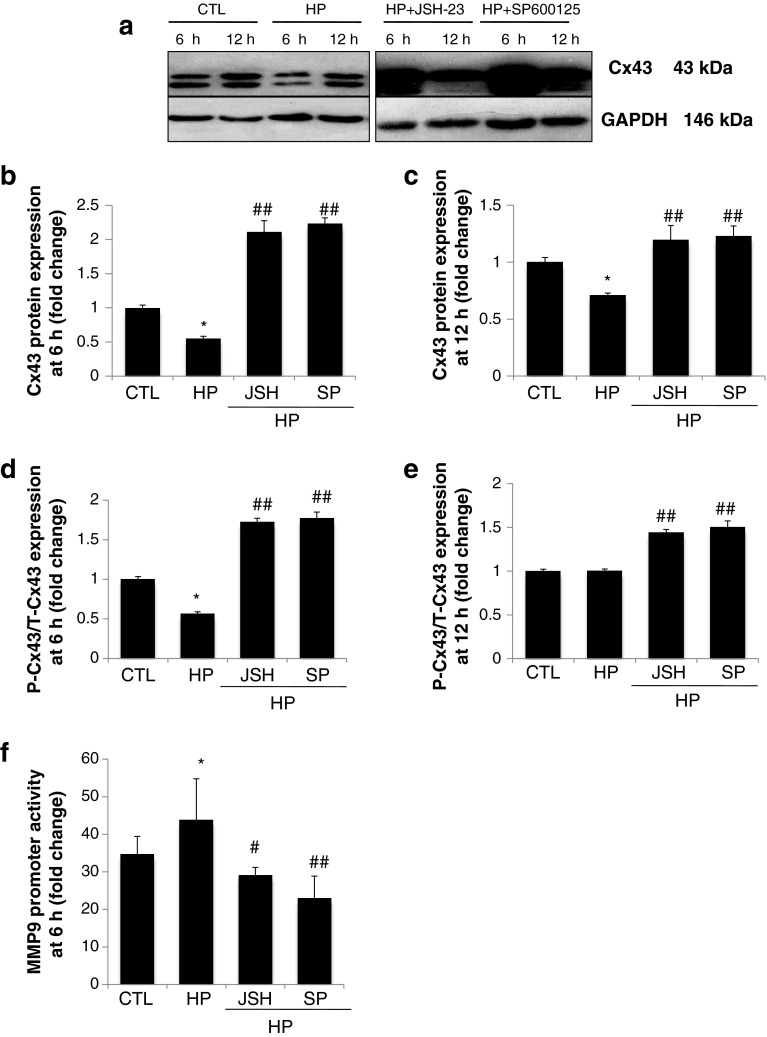
**a** Showing Western Blot band of Cx43 with treatment of hypoxia in the absence or presence of JSH-23 (30 μM) or SP600125 (10 μM) at 6 h and 12 h. **b–f** The quantified results are depicted in the form of bar graphs. Cx43 protein expression with treatment for 6 h (**b**) and 12 h (**c**). Hypoxia induced a decrease in Cx43 expression, however, this effect was notably attenuated by both NF-κB and AP-1/c-Jun inhibitors, JSH-23 and SP600125, respectively. The ratio of P-Cx43 to T-Cx43 is shown in (**d–e**). MMP-9 promoter activity assessed with Dual-Luciferase Reporter Assay System was shown in **f**. Results represent the mean ± SEM of three separate experiments. (*Asterisk*) Significantly different as compared to the control. (*Hash*) data in the groups co-treated with hypoxia and specific inhibitor are significantly different from those treated with hypoxia alone

JNK/SAPK inhibitors significantly reversed the decrease in Cx32 and Cx43 protein expression [19]. To further evaluate the roles of the transcription factors NF-κB and AP-1/c-Jun in the regulation of Cx43, H9C2 cells were cultured in the presence of specific inhibitors of NF-κB or AP-1/c-Jun and total Cx43 protein expression was assessed by Western blot analysis. As indicated in Fig. 4a, Cx43 expression decreased following hypoxia at 6 and 12 h. However, this effect was notably attenuated by both NF-κB and AP-1/c-Jun inhibitors, JSH-23 and SP600125, respectively (Fig. 4b–c). This indicates that NF-κB and AP-1/c-Jun contribute to the regulation of Cx43 expression under hypoxic conditions. Both JSH-23 and SP600125 demonstrated the most obvious effects on Cx43 expression at 6 h. Interestingly, both JSH-23 and SP600125 shifted Cx43 expression to its phosphorylated forms under hypoxic conditions (Fig. 4d–e).

Our previous study indicated that the transcription factors NF-B and AP-1 are involved in the regulation of MMP-9 promoter activity [13]. To determine the roles that the NF-κB and AP-1 pathways play in modulating MMP-9 activity under hypoxic conditions, MMP-9 promoter activity was assessed following treatment with JSH-23 and SP600125. As shown in Fig. 4f, both JSH-23 and SP600125 protected against hypoxia-induced MMP-9 promoter upregulation.

## Discussion

Primary cardiac myocytes are widely used to investigate cellular and molecular changes in cardiac studies; however, the major disadvantage of primary cardiac myocytes is the high number of neonatal animals that are required. The H9C2 rat cardiomyocyte cell line is a reliable alternative for some studies. H9C2 rat cardiomyocytes demonstrate many similarities to primary cardiomyocytes, including membrane morphology, G-protein signaling expression, and electrophysiological properties [20, 21], and were used in all of our experiments.

It is widely accepted that oxidative stress plays a central role in the induction of pathogenic cell responses. The activation of the redox-regulated ERK1/2 and Akt signaling cascades has been observed in atherosclerosis and myocardial ischemia [22]. NF-κB acts broadly on genes that affect cell survival, differentiation, and proliferation. NF-κB is also associated with cancer, autoimmune diseases, and cardiovascular disease [23]. The contribution of NF-κB to cardiovascular disease, like atherosclerosis, is most easily understood in the context of chronic inflammation, wherein proinflammatory cytokines drive the activation of NF-κB that in turn drives target gene expression [24]. Previous studies report that the NF-κB inhibitor BAY11-7082 and the PI3K inhibitor LY294002 both significantly reverse the effects of Toll-like receptors via the ligand-mediated downregulation of Cx43 expression [25]. The nuclear transcription factor AP-1 can combine with many genes and, subsequently, regulate the transcription of these target genes. AP-1 also facilitates the transcription and expression of Cx43 in myometrial cells. Blocking the AP-1 sites that bind to the Cx43 promoter can neutralize the corticotropin-releasing hormone-induced upregulation of Cx43 [26]. Cx43 mRNA expression increases in cardiac myocytes following the addition of amphetamine, and these effects are completely attenuated by the AP-1/c-Jun inhibitor SP600125 [27]. Angiotensin II induces Cx43 expression in vascular smooth muscle cells via the activation of transcription factor AP-1 [28].

**Fig. 5:**
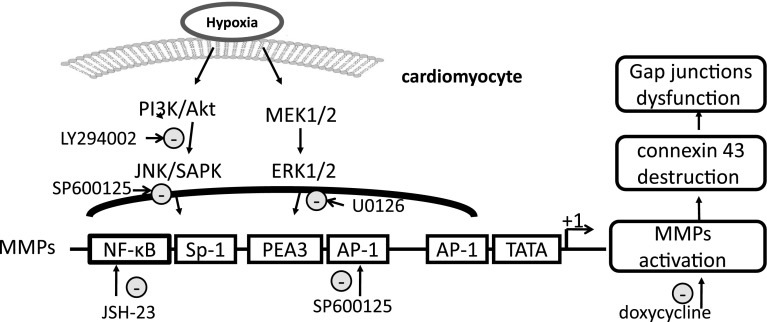
Schematic diagram of the MAPK signaling pathways involved in regulation of Cx43 expression by modulating MMP-9 enzyme. The regulation of Cx43 expression was studied by blocking the pathways of PI3K/Akt or MEK/ERK 1/2 NF-κB or AP-1/c-Jun and MMPs, respectively, with specific inhibitors. The signaling cascades, transcription factors NF-κB and AP-1/c-Jun, and MMPs activity appear to be critical factors in hypoxia-induced disruption of gap junction integrity

To assess the roles of MMPs and MAPK signaling in the regulation of Cx43 gene expression in H9c2 cardiomyocytes, the MMP inhibitor doxycycline, MEK/ERK 1/2 inhibitor U0126, and PI3K inhibitor LY294002 were used in the present study. In agreement with previous data, the inhibition of MMP activity attenuated the hypoxia-induced downregulation of Cx43 protein expression in H9c2 cardiomyocytes (Fig. 2a–d). One novel finding of the present study is that ERK1/2 and PI3K/Akt signaling act differently on Cx43 expression. Inhibiting ERK1/2 signaling attenuated the hypoxia-induced downregulation of Cx43 protein expression, however, blocking PI3K/Akt enhanced the loss of Cx43 in cardiomyocytes (Fig. 2a–c). MAPK signaling probably acts on Cx43 expression by modulating MMP-9 enzymes, as indicated in Fig. 3. Our previously reported study indicated that transcription factors NF-κB and AP-1 are involved in the regulation of MMP-9 promoter activity by mutating the binding sites of NF-κB and AP-1 [13]. To further study the functions of transcription factors under hypoxic conditions, JSH-23 and SP600125 were used to block the NF-κB and AP-1/c-Jun pathways. As shown in Fig. 4a–c, the administration of JSH-23 and SP600125 attenuated the hypoxia-induced loss of Cx43 while also protecting against MMP-9 promoter upregulation (Fig. 4f). This is a good indication that MMP activity, MEK/ERK1/2 and AP-1/c-Jun signaling, and NF-κB play an important roles in regulation of Cx43 expression in H9c2 cardiomyocytes, as summarized in Fig. 5.

The Cx43 expression level depends on the time of oxygen deprivation. Cultured atrial cells exposed to hypoxia for 24 and 48 h demonstrate 75 and 90 % reductions in the Cx43 protein level, respectively [29]. In the present study, exposure to hypoxia for 6 and 12 h caused a decrease in the Cx43 level by approximately 30–50 % (Figs. 2b–c and 4b–c).

Studies indicate that phosphatases may regulate connexin during cardiac ischemia [30]. Connexin phosphorylation is associated with assembly, stability, and other channel properties in gap junctions [31]. p-Cx43 is the major constituent of gap junctions; however, dephosphorylation of Cx43 occurs during hypoxia and ischemia and is associated with p-ERK1/2 levels [32]. Our results are partly consistent with studies in the literature indicating that MAPK signaling affects p-Cx43. Coexposure to hypoxia and U0126, JSH-23, or SP600125 shifts Cx43 expression to p-Cx43; however, the dephosphorylation of Cx43 is induced by the addition LY294002 (Figs. 2d–e, 4d–e).

Nevertheless, it should be noted that these data were collected by a primary study investigating the connection between MAPK signaling, MMP activity, and gap junctions. Further studies should be performed to investigate the exact mechanisms that regulate Cx43, especially the phosphorylated and dephosphorylated forms of Cx43. This will lead to better understanding of the working mechanisms involved in MAPK signaling, MMP, and Cx43 expression.

In summary, exposure to hypoxia decreases Cx43 protein expression in H9c2 cardiomyocytes, inhibits MMP activity, and blocks ERK1/2 signaling, thereby attenuating the hypoxia-induced downregulation of Cx43. Transcription factors NF-κB and AP-1/c-Jun participate in the regulation of hypoxia-induced Cx43 expression. MAPK signaling acts on Cx43 expression, most likely by modulating MMP enzymes.

## Abbreviations

Cx43: Connexin 43
MMPs: Matrix metalloproteinases
MAPK: Mitogen-activated protein kinases
AP-1: Activator protein-1
ECM: Extracellular matrix
PI3K: Phosphoinositide-3 kinase
NF-κB: Kappa-light-chain enhancer of activated B cells
LPS: Lipopolysaccharide
